# Does Lip Augmentation With Hyaluronic Acid Affect Lip Motricity in Young Patients?

**DOI:** 10.1111/jocd.70744

**Published:** 2026-02-25

**Authors:** Victor R. M. Munoz‐Lora, Wagner Baseggio, Pietra Roschel, Lorenna Zillo, Camila Dias, Ana C. N. Carnevali, Victor Rogerio, Patrícia Pauletto, Marcelo Germani

**Affiliations:** ^1^ Department of Facial Aesthetics Guarulhos University São Paulo Brazil; ^2^ HOF Pro Academy Rio Verde, Goiás Brazil; ^3^ Facial Academy Paraná Brazil; ^4^ Department of Biochemistry Federal University of São Paulo, Paulista School of Medicine São Paulo Brazil; ^5^ Private Practice São Paulo Brazil; ^6^ Universidad de Las Americas Quito Ecuador; ^7^ Department of Biological Sciences, Bauru School of Dentistry University of São Paulo Bauru Brazil

**Keywords:** facial aesthetics, facial anatomy, hyaluronic acid, lip filling

To the Editor,

Lip augmentation with hyaluronic acid (HA) fillers is one of the most frequently performed aesthetic procedures worldwide, with 1 449 565 procedures recorded in the United States in 2024 alone [1]. This growing interest, particularly among younger patients without signs of aging, highlights the importance of understanding not only volumetric outcomes but also functional implications related to lip motricity [2]. Despite the increasing popularity of HA fillers, few studies have investigated whether lip augmentation affects essential orofacial movements. The aim of this work is to present new clinical data assessing the impact of HA lip augmentation on lip motricity in young patients, using both objective volumetric measurements and functional tests.

In this clinical study, 20 female volunteers aged 20–30 years underwent lip augmentation using 1 cc of HA (Rennova Lips, Rennova, Brazil). A 27 G needle (TSK, Tochigi, Japan) was attached to the HA syringe and inserted vertically into each anterior intralabial injected into the anterior intralabial [3] compartments following a standardized protocol [2]. Volumetric assessment with 3D stereophotogrammetry (3D‐SQ) was performed at baseline, immediately after treatment, and at 30 and 90 days. Motricity was evaluated at baseline, 30, and 90 days using two functional tests: whistling and drinking through a straw, both quantified using a visual analogue scale. Data were analyzed using MANCOVA and repeated‐measures ANOVA to assess the effects of time and volume on labial motricity and lip volume after HA injections.

Our findings confirm a significant volumetric enhancement immediately after HA injection, with a gradual and expected decrease over the 90‐day period (Figure 1A). For the whistle test, the mean motricity scores were 9.60 (SD ± 0.598) at baseline, 9.50 (SD ± 1.15) at 30 days, and 9.53 (SD ± 0.964) at 90 days. For the drinking water with a straw test (DWWS), the mean scores were 9.15 (SD ± 2.18) at baseline, 9.50 (SD ± 1.24) at 30 days, and 9.63 (SD ± 0.895) at 90 days (Figure 1B). Volumes remained significantly higher compared to baseline throughout follow‐up. Importantly, the functional assessments demonstrated that lip motricity was preserved at all time points (Figure 2A,B). Neither time nor volume variations influenced motricity scores, as shown by multivariate analyses.

**FIGURE 1:**
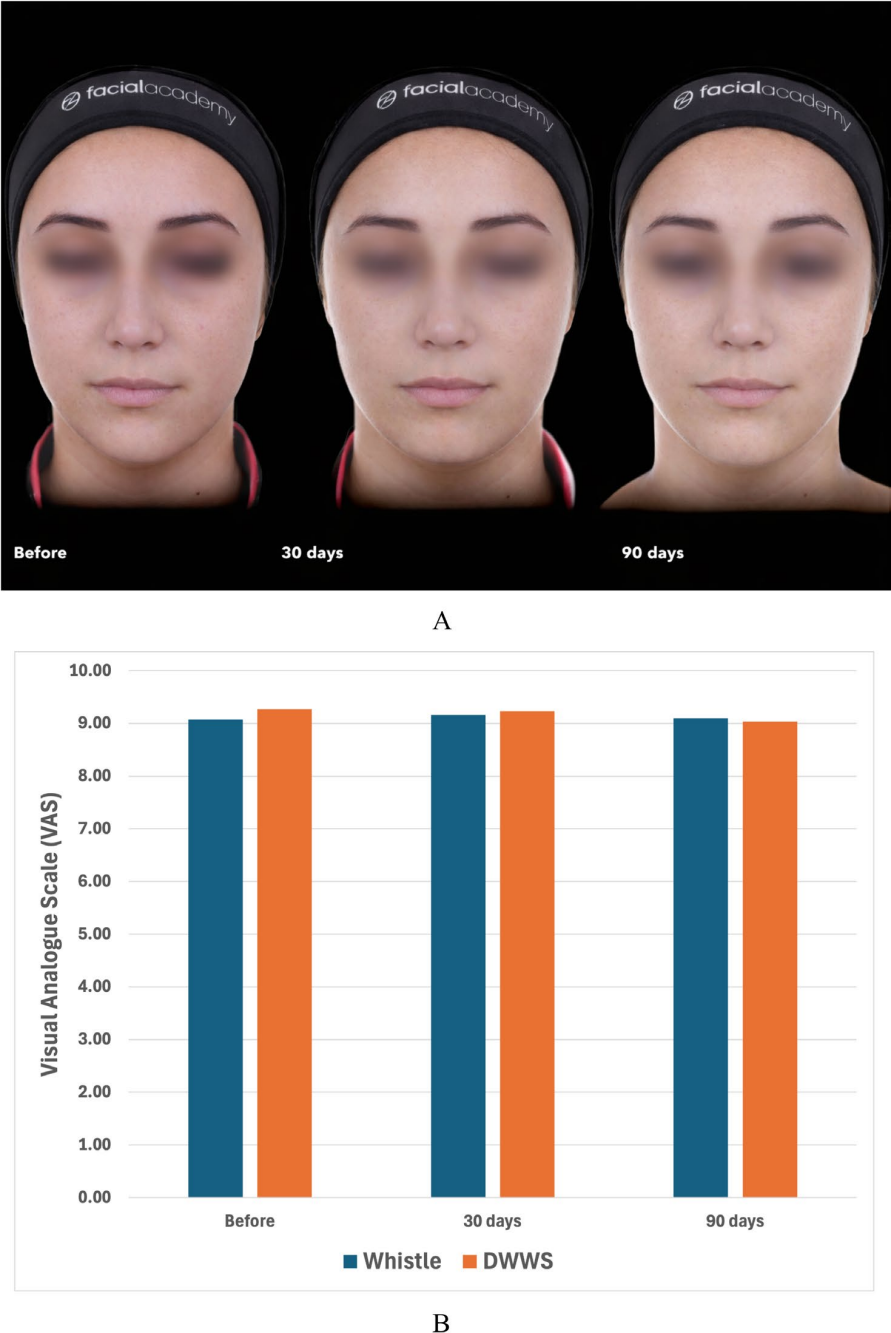
(A) A 24‐year‐old patient before, 30 days and 90 days after lip augmentation with hyaluronic acid. (B) Functional motricity tests (whistle test and drinking water with straw test—DWWS) measured with a visual analogue scale (VAS) before, 30 days, and 90 days after treatment. No significant differences were found among time.

**FIGURE 2:**
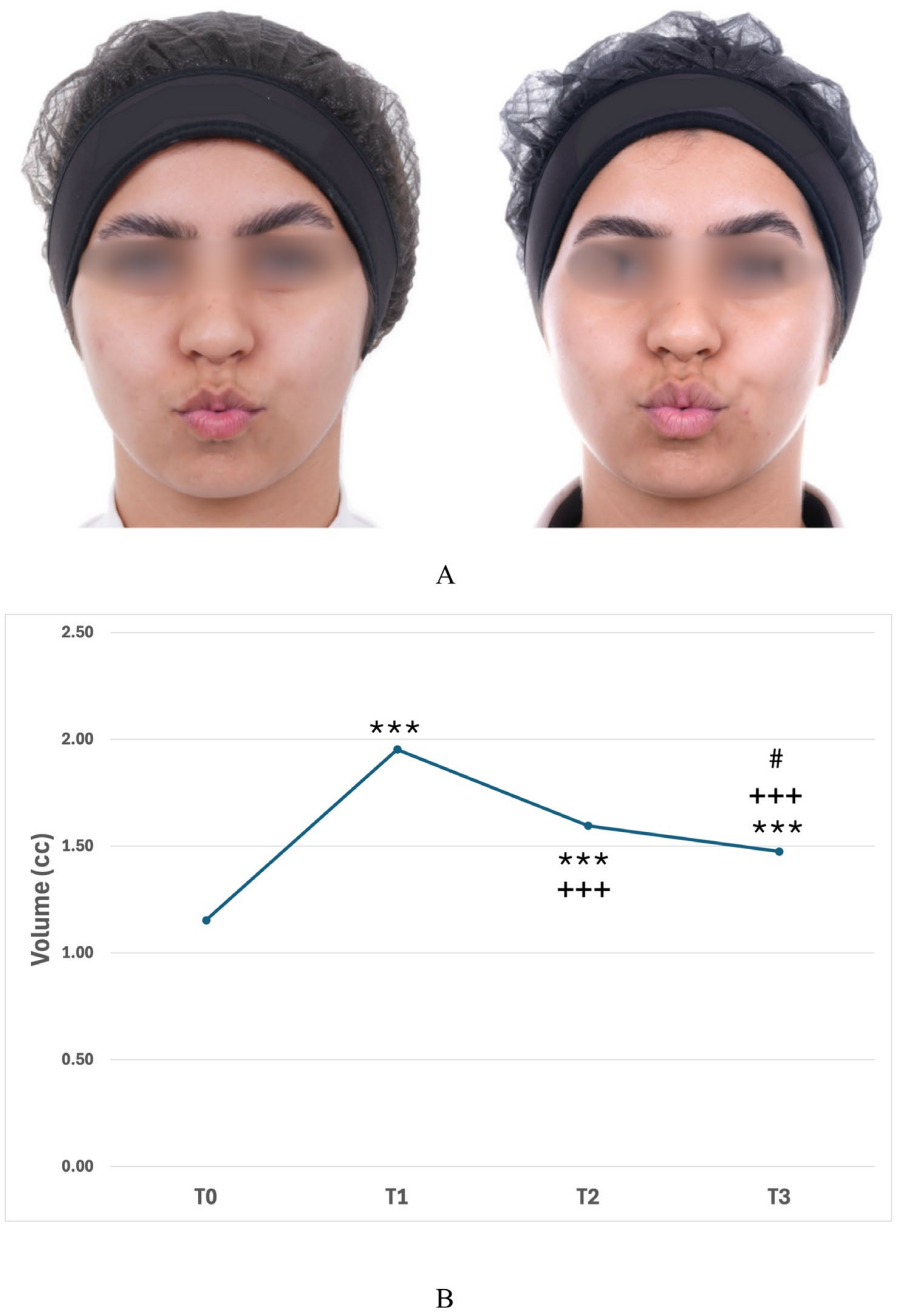
(A) Female patient, 27 years old, before (left) and 30 days after (right) lip augmentation with hyaluronic acid, performing lip protrusion (“kissing” movement). Labial motricity remained preserved. (B) Labial volume (cc) among time (T0—baseline; T1—immediately after; T2–30 days; T3–90 days) lip augmentation with hyaluronic acid. Mean ± SE. (***) = *p* < 0.001 vs. T0; (+++) = *p* < 0.001 vs. T1; (#) = *p* < 0.05 vs. T2 (Repeated measures ANOVA followed by Tukey's multiple comparisons test).

These findings complement prior work by Bertucci et al. [4] and Nikolis et al. [5], who also reported high satisfaction and preserved dynamics after lip augmentation, although without specifically assessing functional motricity through standardized tests. Our study adds to the literature by providing a direct functional assessment, reinforcing that HA augmentation when anatomically guided and properly distributed can enhance lip volume without impairing dynamic performance [3].

Additionally, patient satisfaction at 90 days was high (mean 81.4 on a 0–100 scale), which may reflect both aesthetic improvement and preserved functionality. It is essential to highlight that young patients, particularly those frequently exposed to unrealistic aesthetic standards on social media, often have elevated expectations. The combination of increased volume, maintained mobility, and low adverse event rates likely contributed to the positive perceptions reported.

We acknowledge limitations, including the small and restricted sample size, the homogeneous population of young female participants, and the absence of a control group, although the latter is ethically challenging in aesthetic interventions. Additionally, the relatively short follow‐up period and the use of a single HA product and standardized technique may limit the generalizability of the findings. Nonetheless, these preliminary results provide clinically relevant evidence supporting the safety and functional preservation associated with anatomically guided HA lip augmentation.

Hyaluronic acid lip augmentation increases lip volume without impairing motricity when anatomically guided injections are used, preserving function even in young patients. Further studies with larger samples are warranted.

## Author Contributions

The authors V.R.M.M.‐L. and M.G. contributed to developing the concept for the work; acquiring, analyzing, and interpreting the data; drafting the article; and revising it critically for important intellectual content. They also granted final approval to the version to be published and agreed to be accountable for all aspects of the work in ensuring that questions related to the accuracy or integrity of any part of the work were appropriately investigated and resolved. The authors W.B., P.R., L.Z., C.D., A.C.N.C., V.R., and P.P. contributed to the acquisition and interpretation of data and critically revised the article for important intellectual content.

## Funding

The hyaluronic acid used in this study was generously provided by Rennova, Innovapharma Brasil Farmaceutica Ltda, Goiania, Brasil. However, the company was not involved in the study design, data collection, analysis, or interpretation of the results.

## Ethics Statement

This clinical study was approved by the Research Ethics Committee of Centro Universitário Católico Salesiano Auxilium—UniSalesiano/SP (Approval number: 6.657.993; CAAE: 74170823.8.0000.5379) on February 20, 2024. All procedures were conducted in accordance with the Declaration of Helsinki and relevant national regulations.

## Consent

Written informed consent was obtained from all participants prior to their inclusion in the study. All participants were informed about the potential risks and benefits of the procedure and were guaranteed confidentiality throughout the data collection and publication process.

## Conflicts of Interest

The authors declare no conflicts of interest.

## Data Availability

The data that support the findings of this study are available from the corresponding author upon reasonable request.
